# Complementary Feeding: From Tradition to Personalized Nutrition

**DOI:** 10.3390/children11010080

**Published:** 2024-01-09

**Authors:** Raffaella Panza, Maria Elisabetta Baldassarre

**Affiliations:** Department of Interdisciplinary Medicine, University of Bari Aldo Moro, Piazza Giulio Cesare 11, 70124 Bari, Italy; raffaella.panza@policlinico.ba.it

The introduction of solid foods into the infant’s diet is a moment of great change in the routine of parents and children. In recent years, weaning has been progressively “liberalized”, eliminating, for example, the practice of timed insertions. However, such freedom easily translates into a lack of references for parents, who are too often given imprecise—if not even conflicting—indications deriving from various sources (pediatricians, friends, the internet, books and magazines on the subject).

In the present Special Issue, we focused on the concept of personalized nutrition in all stages of life, and we investigated the currently most common weaning practices.

What is known is that parents and caregivers play a fundamental role in the weaning process; therefore, they should be correctly supported by reference figures. Nutritional deficits and excesses are all potentially avoidable if parents are correctly informed. Conversely, the lack of nutritional knowledge of diets and pediatrician supervision may be responsible for the increased risk of serious nutritional deficiencies, as well as overweight and obesity. Even though many recommendations are available [1,2,3], there are substantial variations in complementary feeding modalities between countries due to different traditions. Moreover, alternative weaning strategies, such as vegetarian/vegan weaning or baby-led weaning, are increasingly common among families [4]. In this context, the shortage of evidence-based data on this topic creates confusion, which translates into different behaviors among both pediatricians and families.

Special attention should be paid to preterm infants, a more vulnerable population, often affected by extrauterine growth retardation. A recent position statement by Italian neonatal and pediatric societies [5] suggests that complementary feeding in preterm infants should be started at 5–8 months of chronological age as long as neurodevelopmental skills allow for the consumption of solid foods (which usually occurs starting from 3 months’ corrected age). As for the type of foods and the sequence and speed of introduction, the same guidelines available for term infants should also be applied for ex-premature infants. Moreover, it seems unnecessary to delay the start of weaning in preterm infants to prevent overweight and obesity, as the timing of complementary feeding in this population is unrelated to the incidence of such diseases in childhood and adulthood.

Notably, parents and caregivers should learn to interpret their child’s hunger and satiety signals, encouraging a mutual relationship in which the child is free to experiment without forcing. The general parental educational style and “responsive” food care practices are capable of significantly influencing the development of eating behavior. A responsive/authoritative parenting style contributes to the development of “secure attachment” in childhood and has been associated with various positive effects on children. A systematic review of 36 studies highlighted that children with “authoritative” parents have healthier eating behaviors; are physically more active; and have lower BMI values than the children of parents who adopt an authoritarian, indulgent or permissive educational model [6].

Responsive feeding comprises a set of ready, contingent, emotionally and developmentally appropriate responses by the caregiver to the child’s hunger and satiety signals; the degree of congruence with which this is achieved can strengthen or hinder the child’s ability to self-regulate, a basic element of responsive nutrition.

Parents often need to gain greater confidence in their child’s ability to self-regulate how much to eat by applying Satter’s principle of division of responsibility “parent provides... child decides” [7]; according to this principle, parents have the responsibility of offering healthy foods and structuring appropriate meal times and methods to ensure that the appetite–satiety cycle occurs regularly, while the child has the responsibility to decide freely, without parental interference, whether and how much to eat.

In conclusion, weaning represents an important developmental milestone with short- and long-term health effects. Pediatricians should be able to provide the right answers to parents’ requests and explain the pros and cons of various weaning methods to ensure the best growth and health of children. Weaning based on “responsive feeding” should be encouraged. During breastfeeding and infancy, as well as during pregnancy, correct information from an expert pediatrician or nutritionist is essential to improve nutritional care, monitor any nutritional deficiencies and prescribe supplements if necessary.

## Data Availability

All relevant data are included in the manuscript.
